# Assessing Parent Decisions About Child Participation in a Behavioral Health Intervention Study and Utility of Informed Consent Forms

**DOI:** 10.1001/jamanetworkopen.2020.9296

**Published:** 2020-07-31

**Authors:** Stephanie A. Kraft, Kathryn M. Porter, Devan M. Duenas, Erin Sullivan, Maya Rowland, Brian E. Saelens, Benjamin S. Wilfond, Seema K. Shah

**Affiliations:** 1 Treuman Katz Center for Pediatric Bioethics, Seattle Children’s Research Institute, Seattle, Washington; 2 University of Washington School of Medicine, Department of Pediatrics, Seattle; 3 Seattle Children’s Core for Biomedical Statistics, Seattle Children’s Research Institute, Seattle, Washington; 4 Center for Child Health, Behavior and Development, Seattle Children’s Research Institute, Seattle, Washington; 5 Mary Ann & J. Milburn Smith Child Health Research, Outreach, and Advocacy Center; Stanley Manne Children’s Research Institute; Ann & Robert H. Lurie Children’s Hospital of Chicago, Chicago, Illinois; 6 Department of Pediatrics, Northwestern University Feinberg School of Medicine, Chicago, Illinois

## Abstract

**Importance:**

Obtaining informed consent is an important ethical obligation for clinical research participation that is imperfectly implemented. Research on improving consent processes often focuses on consent forms, but little is known about consent forms’ influence on decision-making compared with other types of engagement.

**Objective:**

To evaluate whether parents decide whether to enroll their children in research before or after they receive the consent form.

**Design, Setting, and Participants:**

An online survey of 88 parents who enrolled or declined to enroll their child in a weight management intervention study between January 2, 2018, and June 24, 2019, was conducted; surveys were completed between February 2, 2018, and July 9, 2019. A 31-item survey asked about impressions of the study throughout the enrollment process, timing of enrollment decisions, and decision-making factors. Responses were summarized descriptively and subgroups were compared using the Fisher exact test or χ^2^ test.

**Main Outcomes and Measures:**

Self-reported timing of enrollment decision.

**Results:**

A total of 106 parents were approached and gave permission for their contact information to be shared with the study team; 22 additional parents declined to allow their information to be shared, and 24 lost contact with the partner study before they could be asked for permission. A total of 88 parents (67 enrollees, 21 decliners) completed the survey (83% participation rate); 79 of 88 reporting gender (instead of sex, as biological sex was not relevant to survey) information were women (91%), 66 participants (75%) were non-Hispanic White, and 63 participants (72%) had annual household incomes greater than or equal to $70 000. No significant differences in respondent characteristics between enrollees and decliners were identified. Fifty-nine parents (67%) responded that they decided whether to enroll in the weight management study before receiving the consent form. Only 17 of 69 parents (25%) who remembered receiving the consent form responded that it taught them new information.

**Conclusions and Relevance:**

The findings of this study suggest that interventions to improve informed consent forms may have limited influence on decision-making because many research decisions occur before review of the consent form. It appears that regulatory review and interventions to improve decision-making should focus more on early engagement (eg, recruitment materials). Future studies should test timing of decisions in other types of research with different populations and clinical settings.

## Key Points

**Question:**

Is the timing of parents' decision on whether to enroll their children in research associated with review of the informed consent form?

**Findings:**

This online survey study of 88 parents who either enrolled (n = 67) or declined to enroll (n = 21) their child in a weight management intervention study revealed that 67% decided whether to enroll before receiving the consent form. Twenty-five percent who remembered receiving the consent form reported that it taught them new information.

**Meaning:**

The findings of this study suggest that regulatory review and interventions to improve decision-making should focus on early engagement throughout the recruitment and consent process rather than just consent forms to affect decision-making.

## Teaser

This survey study examines use of the informed consent form in decision-making by parents on enrollment of their children in a weight management study.

## Introduction

Obtaining informed consent is widely considered a critical ethical obligation for clinical research participation.^1^ The consent process serves several functions, including protecting participants, respecting their autonomy, and achieving transparency.^2^ During this process, prospective participants may have multiple interactions with the study team, as well as the opportunity to review an informed consent form describing the study’s procedures, risks, benefits, and alternatives.^3^ Much research has focused on the consent form rather than other aspects of the consent process, assuming that interventions to improve consent forms can increase individuals’ understanding of and satisfaction with research.^4^ Despite research to design interventions to improve consent forms, these studies have demonstrated only limited success at improving participant understanding, satisfaction, or quality of decision-making.^5^

One hypothesis to explain why interventions to improve consent forms have had scant success is that many individuals decide whether to enroll in research relatively early, before reading a consent form. Aspects of recruitment and enrollment that occur before consent, including interactions with research teams, review of recruitment materials, and early refusals to participate, have been understudied.^6^ Our hypothesis that many research decisions occur before review of the consent form is supported by data suggesting that participants tend not to read consent forms thoroughly before enrolling^7,8^ and the findings of intervention studies making major modifications to the consent form with no difference noted in understanding or satisfaction.^9,10^ Despite this growing body of evidence about the importance of research engagement outside of consent forms, federal research regulations call for detailed institutional review board (IRB) oversight of the content of consent forms but give little attention to the rest of the recruitment and enrollment process.^11^

Better understanding of the timing and process of decision-making, among both study enrollees and decliners, is needed to develop interventions to enhance the quality of research decisions. We sought to address this gap in the literature and test our hypothesis that many people decide whether to enroll in research before reviewing the consent form by surveying parents who enrolled or declined to enroll their child in a family-based pediatric weight management intervention study about when, how, and why they made their decisions. We selected this partner study because it involved a multipart enrollment process, which allowed us to specifically examine decision-making timing. In addition, because the study involved children whose parents made decisions on their behalf, we expected decision-making to be more careful and deliberative than in adults deciding for themselves, which would yield a conservative estimate of whether and how much early decision-making occurs.

## Methods

We emailed an online survey to parents who had either enrolled or declined to enroll their child in a behavioral intervention study between January 2, 2018, and June 24, 2019. Surveys were completed between February 2, 2018, and July 9, 2019. This study was determined to be exempt from review by the Seattle Children’s Hospital institutional review board per regulations found at 45 CFR 46.101(b)(2) (pre-2018 requirements) (use of survey procedures). This study followed the American Association for Public Opinion Research (AAPOR) reporting guideline for survey studies.

**Figure 1.**
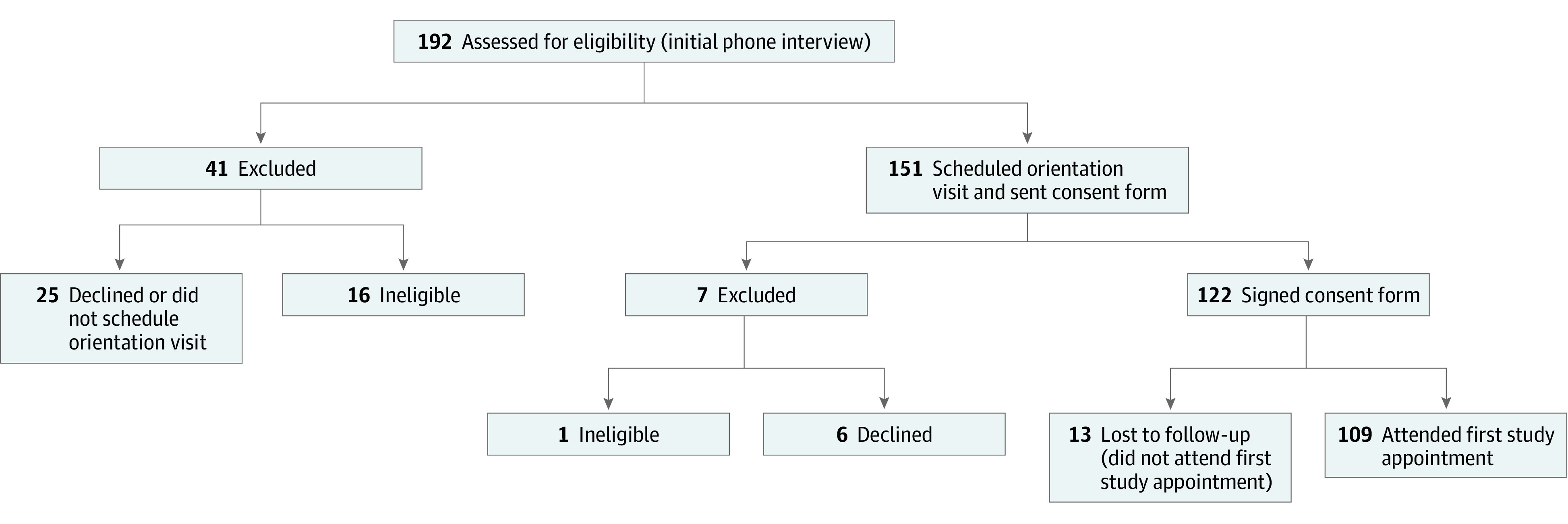
Success in Health: Impacting Families Together (SHIFT) Recruitment Flowchart, January 2, 2018, to June 24, 2019 Recruitment, screening, and consent for the SHIFT study during the time of survey recruitment.

We partnered with a pediatric weight management intervention study called Success in Health: Impacting Families Together (SHIFT). The goal of the SHIFT study was to evaluate the outcome of professional vs peer-based delivery of a behavioral intervention to improve eating and activity behaviors for better weight management in children aged 7 to 11 years. The Box details the SHIFT recruitment and enrollment process. Figure 1 shows the SHIFT enrollment flowchart during the time that we administered our survey. Study recruitment was ongoing, but study start dates were staggered and families enrolled in cohorts, approximately twice a year, at 2 to 3 sites. We surveyed participants who were recruited in spring 2018, fall 2018, and spring 2019.

Box. **Stages and Details of the Success in Health: Impacting Families Together (SHIFT) Enrollment Process**

**Recruitment Flyers**

- Mailed to families with children in the eligible age range near study clinic sites
- Distributed to pediatric and other primary care physicians for distribution to families
- Briefly described eligibility criteria, study activities and locations, and financial incentives
- Interested families completed brief online survey or contacted study team to determine eligibility

**Initial Phone Interviews**

- Study staff further assessed eligibility and interest
- Staff scheduled eligible and interested families for orientation visit
- After interview, staff mailed families consent form with instructions to review and discuss with child

**Consent Form**

- Study procedures, including intervention and assessments
- Potential risks/burdens: hunger, soreness/injury from being more active, inconvenience/time commitment, family disagreements, and breach of confidentiality
- Potential benefits: healthier weight status, increased activity and healthier diet, and better overall health and mood

**In-Person Orientation Visit**

- Presentation about the study that closely followed the consent form
- Height and weight measurements to confirm eligibility
- Eligible families received second copy of consent form to review and sign

**Review and Signing of Consent Form**

- Staff member reviewed consent form and answered questions
- Accompanying 4-page assent form and 1 to 2 questions asked to assess child's comprehension
- Opportunity to meet privately as a family and/or with study staff

**Study Activities and Compensation**

- 20 Weekly treatment visits (90 minutes each)
- Pretreatment and posttreatment, as well as 6- and 12-month follow-up, assessments (60-90 minutes each)
- Gift card incentives for posttreatment and follow-up assessments totaling $125
- Eligible for up to $200 in financial help with childcare and $200 for travel

We developed a 31-item survey to administer to parents who had enrolled or declined to enroll their child in the SHIFT study (eAppendix in the Supplement). Survey questions addressed (1) how parents learned about SHIFT, (2) first impressions of the study, (3) the SHIFT orientation visit and presentation, (4) the SHIFT consent process and form, (5) the timing and process of the enrollment decision, and (6) demographics. We completed cognitive interviews with 2 SHIFT enrollees and 2 decliners before administering the survey to the full sample, using the think-aloud approach to assess understandability of the questions.^12^ We subsequently revised the instrument for clarity.

SHIFT study staff informed parents about our survey after the parents decided on whether to enroll in SHIFT and asked the parents’ permission to provide us their contact information. Enrollees included individuals who returned a signed consent form to join the upcoming cohort, even if they did not later participate in all study activities. Decliners included individuals who chose not to join SHIFT at any point between the initial phone interview and review of the consent form at the end of the orientation visit. We emailed SHIFT enrollees and decliners a link to the survey, followed by up to 2 reminder emails. Respondents received a $10 gift card incentive. Survey data were collected and managed using REDCap electronic data capture tools hosted at the University of Washington.^13^

### Statistical Analysis

Ninety-five respondents submitted full or partial responses to the survey; of these, 7 responses were excluded and 88 were included in the final analysis. Three surveys were excluded owing to insufficient data and 2 because they were repeats of previous respondents. There were also 2 survey participants who initially responded as decliners in one cohort but enrolled in SHIFT in a subsequent cohort and completed the survey again. Because their responses as enrollees could not be viewed as comparable to those exposed to the consent process for the first time, their second surveys were excluded. All survey responses were summarized descriptively. Comparisons between subgroups (enrollees vs decliners; those who decided before contact with the study team vs after the initial phone interview but before the orientation visit vs after receiving the consent form, ie, during or after the orientation visit) were made using the Fisher exact test or χ^2^ test, and an α level of .05 was used for significance testing, which was 2 sided and unpaired. Statistical analysis was conducted using SAS, version 9.4 (SAS Institute Inc).

## Results

### Respondent Characteristics and Decisions

Based on self-reported responses, the 88 survey respondents were primarily women (91%), aged 26 to 49 years (85%), non-Hispanic White (75%), married (75%), at least college educated (65%), and with an annual household income greater than or equal to $70 000 (72%). Only 14 parents (16%) had participated in research before (Table). These 88 respondents of 106 parents of SHIFT enrollees and decliners (72 enrollees, 34 decliners) invited to take our survey represented an 83% participation rate overall.^14^ Respondents included 67 SHIFT enrollees (76% [93% participation rate]) and 21 decliners (24% [62% participation rate]). Although our participation rate was higher for enrollees than decliners, the participation rate for decliners was over 60%. There were no significant demographic differences between enrollees and decliners.

**Table.** Demographic Summary by Timing of Decision and Enrollment Status

Characteristic	No. (%)							
	Overall	Timing of decision				Enrollment decision		
	Respondents, % (n = 88)	Before any contact with study team (n = 27)	After initial phone contact, before orientation (n = 32)	After receiving consent form or other (n = 29)	*P* value^a^	Enroll (n = 67)	Decline (n = 21)	*P* value^a^
Gender^b^								
Male	8 (9)	3 (11)	3 (9)	2 (7)	.90	5 (8)	3 (14)	.39
Female	79 (91)	24 (89)	29 (91)	26 (93)		61 (92)	18 (86)	
Age, y								
26-49	75 (85)	24 (89)	27 (84)	24 (83)	.87	57 (85)	18 (86)	>.99
50-64	13 (15)	3 (11)	5 (16)	5 (17)		10 (15)	3 (14)	
Race/ethnicity								
Non-Hispanic White	66 (75)	20 (74)	26 (81)	20 (69)	.53	48 (72)	18 (86)	.26
Other	22 (25)	7 (26)	6 (19)	9 (31)		19 (28)	3 (14)	
Highest educational level								
Did not graduate college	31 (35)	9 (33)	8 (25)	14 (48)	.16	27 (40)	4 (19)	.12
College degree or higher	57 (65)	18 (67)	24 (75)	15 (52)		40 (60)	17 (81)	
Total annual household income, %								
<$70 000	22 (25)	12 (44)	5 (16)	5 (17)	.02	19 (29)	3 (14)	.36
≥$70 000	63 (72)	13 (48)	26 (81)	24 (83)		46 (69)	17 (81)	
Prefer not to answer	3 (3)	2 (7)	1 (3)	0		2 (3)	1 (5)	
Marital status								
Married	66 (75)	18 (67)	27 (81)	22 (76)	.60	48 (72)	18 (86)	.41
Divorced or separated	10 (11)	5 (19)	3 (9)	2 (7)		8 (12)	2 (10)	
Never married	12 (14)	4 (15)	3 (9)	5 (17)		11 (16)	1 (5)	
Prior research participation^b^								
Yes	14 (16)	5 (19)	6 (19)	3 (10)	.51	9 (14)	5 (24)	.54
No	67 (77)	21 (78)	24 (78)	22 (76)		52 (79)	15 (71)	
Do not remember	6 (7)	1 (4)	1 (4)	4 (14)		5 (8)	1 (5)	

^a^ Fisher exact test or χ^2^ test.

^b^ Gender was reported on the response form instead of sex, as biological sex was not relevant to survey. Eighty-seven responses (ie, 1 observation missing).

**Figure 2.**
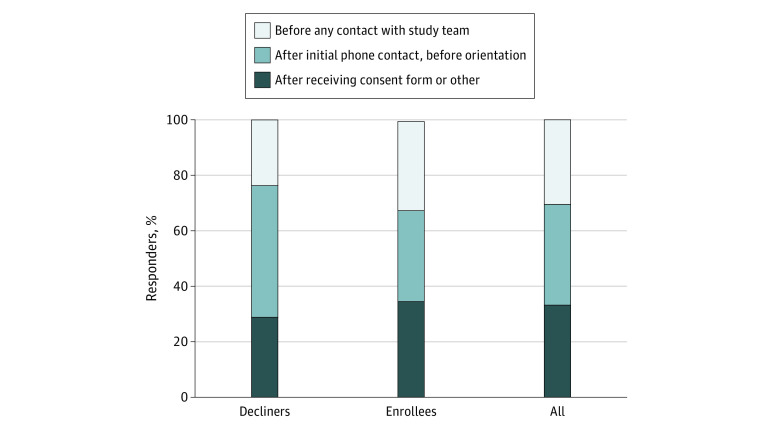
Timing of Enrollment Decision

Fifty-nine of the respondents (67%) reported that they decided whether to enroll their child in SHIFT before receiving the consent form. Of these, 27 respondents (31%) decided after initially hearing about the study and before talking with the study team, and 32 individuals (36%) decided after talking with study staff in the initial phone interview. The remaining 29 parents (33%) decided at some point after they received the consent form by mail, including during or after the orientation visit or at some other time. Only 3 individuals (3%) decided after reviewing the consent form at the end of the orientation visit in the presence of study staff (Figure 2).

We compared respondents based on timing of the decision and found significant differences in income level based on timing of decision (*P* = .02); a larger proportion of those deciding whether to participate before any contact with the study team had annual incomes below $70 000 (12 of 27 [44%]; 95% CI, 25%-65%) compared with those deciding after the phone interview (5 of 32 [16%]; 95% CI, 5%-33%) or after receiving the consent form (5 of 29 [17%]; 95% CI, 6%-36%).

Most respondents heard about SHIFT from a flyer in the mail (64 [73%]) or their child’s physician (11 [13%]). Nearly all respondents (87 [99%]) initially thought it sounded like a good idea to participate. Forty-one parents (47%) sought additional information about the study from a source outside of the study team.

Seventy respondents reported having attended an orientation visit (70 of 87 [80%], owing to 1 missing observation), including all 67 enrollees and 3 decliners (15%, of 20, owing to 1 missing observation). Most of these respondents were somewhat (41 [59%]) or very (27 [39%]) certain that they would enroll when they first arrived at the orientation visit. Those who decided before any contact with the study team were more likely to report being very certain (15 of 22 [68%]) compared with those deciding after the phone interview (10 of 22 [45%]) or those deciding after receiving the consent form (2 of 26 [8%]) (*P* < .001). Most reported that they paid very much attention to the presentation at that visit (63 [90%]) and that it taught them something new about the study (52 of 69 [75%], with 1 missing observation). Thirty-one respondents (44%) reported that the presentation made them more interested in participating and 35 individuals (50%) indicated that the presentation did not change their interest.

Sixty-nine respondents (78%) reported that they recalled receiving a consent form, including 64 enrollees (96%) and 5 decliners (24%). Fifty-seven of these respondents (83%) responded that they read most or all of the consent form, 65 individuals (94%) read the form somewhat or very carefully, and 65 individuals (94%) found the form somewhat or very helpful. However, only 17 parents (25%) indicated that the consent form taught them something new about the study. We found no significant differences in views on the consent form based on enrollment decision or timing.

Most respondents indicated that they liked (65 of 85 [76%], with 3 missing observations) and trusted (62 [70%]) the SHIFT study team member who initially talked with them on the phone very much. Of the 70 respondents who attended the orientation visit, most also responded that they liked (48 [69%]) and trusted (50 [71%]) the orientation study staff very much. Compared with decliners, enrollees were more likely to report very much liking the team member they spoke with on the phone (53 of 64 parents, with 3 observations missing [83%] vs 12 of 21 decliners [57%], *P* = .03). There were no significant differences in these measures based on timing of decision.

### Reasons for Decision

Respondents were initially interested in the study because they hoped it would help them or their child (84 [96%]), wanted to learn from it (47 [53%]), perceived the risks as low (30 [34%]), wanted to advance science (30 [34%]), wanted to help others with childhood obesity (17 [19%]), and were interested in financial incentives (5 [6%]). When asked what they initially disliked about the study, 38 respondents (43%) reported that they did not dislike anything, 31 respondents (35%) reported that the time or location was inconvenient, 7 respondents (8%) thought it required too much time and effort, 5 respondents (6%) were unsure whether it would help, and 4 respondents (5%) were worried that the study could cause harm. Compared with decliners, enrollees were more likely to report that there was nothing they disliked when they first learned about the study (52%; 95% CI, 40%-64% vs 14%; 95% CI, 0%-29%; *P* = .002) and less likely to find the time or location inconvenient (25%; 95% CI, 15%-36% vs 67%; 95% CI, 47%-87%; *P* = .001). Compared with those making their decision later in the process, respondents deciding whether to participate before any contact with the study team were more likely to express interest in financial incentives (19%; 95% CI, 4%-33% vs 0%; 95% CI, 0%-0%; *P* = .002).

Some respondents (31 [35%]) indicated that the most important factor in their decision-making was talking with their child, followed by the research staff (24 [27%]) or a spouse or trusted friend (15 [17%]). Only 3 respondents (3%) indicated that the consent form was most important.

## Discussion

Our findings suggest that the informed consent form did not play a major role in most enrollment decisions in a family-based pediatric weight management study. On the contrary, one-third of the respondents reported that they decided shortly after hearing about the study without having any contact with the study team, and another third decided after having an initial phone conversation with study staff. Very few reported that they decided after reviewing the consent form at the end of the orientation visit. Although most prospective participants indicated that they read the consent form thoroughly and carefully and found it valuable, the form itself did not appear to have been a major factor in their decisions and did not necessarily teach them anything new.

Although our findings may not generalize to all types of studies, these results raise 3 key points for those considering how to improve research decision-making: (1) some individuals may make decisions using heuristics rather than deliberative weighing of the details involved in a trial, (2) regulatory review of research should focus more on early engagement with prospective participants, and (3) financial motivations may drive early decision-making for some individuals.

First, our finding that most respondents reported having decided before reviewing the consent form and often shortly after hearing about the study even before any contact with the study team suggests that individuals may not deliberate about the specifics of a study in the way that a standard decision-making model might assume. This finding aligns with previous work suggesting that decisions about research participation are sometimes based on heuristics.^15,16^ That is, some individuals may use decision-making shortcuts based on factors such as previous views about the study intervention, initial impressions of the research, and trust in the institution or staff. For example, one study identified the affect heuristic, whereby individuals make decisions based on an initial emotional reaction, as a driver of research decisions among adolescents.^15^ Christofides and colleagues^16^ argue that heuristics can be a valuable tool in highly complex situations where there is no optimal solution to the question at hand, such as whether to enroll in research.

In our survey, nearly all respondents were initially positive about the study and most liked and trusted the research staff, suggesting that parents had a positive affective response to the study and staff. Those who ultimately enrolled were more likely to report very much liking the study staff on the first phone call; these initial impressions may have carried through to their decisions. In contrast, decliners, who were more likely to note the study’s inconvenience, may have changed their mind after considering the study’s burdens. Enrollees similarly may have carefully considered the information they received after making an initial decision, yet, unlike decliners, found that the information supported their choice and therefore reported having decided at the earlier time point. While our survey was not designed to fully examine the decision-making process, at a minimum these findings illustrate that a range of factors may influence research decisions and support conceptualizing consent as a relational and context-dependent process.^17,18^

Second, because our findings suggest that at least initial decision-making often occurred before review of the consent form, our study raises questions about the value of refocusing regulatory oversight not just on informed consent forms, but on other aspects of engagement as well. Although respondents reported finding the consent form helpful, other factors may have played a larger role in their decisions, such as prior relationships with the physician who may have alerted them to the study, recruitment materials, phone/email interactions with study staff, internet searches, and conversations with others, all of which are underexplored in the literature and seldom reviewed by IRBs to the same extent as consent forms.^19,20^ For research teams, this recognition of factors outside the consent form builds on prior work similarly highlighting other important aspects of the enrollment process^21,22^ to suggest that interventions to improve prospective participants’ understanding and decision-making should focus more on early stages of recruitment and engagement, including interactions between research teams and prospective participants. Likewise, IRB review could focus more on recruitment materials and the less-tangible aspects of consent processes than has traditionally been done; future research should assess different ways to design recruitment materials, with the goal of providing guidance regarding best practices for recruitment to support well-informed decision-making. Institutional review boards could also address the importance of personal interactions by, for example, ensuring that all members of the research team have guidance and training in best practices for interacting with participants tailored to their role and the study’s context. The importance of a virtuous investigator has long been recognized,^23,24^ but it has been less appreciated that the same holds true for other members of the research team who interact with prospective participants and have the potential to have an influence on their enrollment decisions.^25^

Third, we found that respondents who decided on whether to enroll before any contact with the study team were more likely to indicate that the financial incentives motivated their interest in the study. This group was also more likely to have annual incomes less than $70 000, which is below the 2017 median income ($83 571) in the county in which SHIFT took place.^26^ These findings may also reflect a lack of access to this type of intervention outside of the study. Income was the only demographic characteristic that we found to differ significantly between the groups who made decisions at different times, and we did not have a specific hypothesis about which differences we would find or in what directions. In addition, only a small number of respondents were motivated by financial incentives. This finding should be considered preliminary, requiring further exploration. While financial motivations and limited alternatives do not preclude careful decision-making, these findings support further research into whether participants who may be drawn in by a study’s incentives also have the opportunity to carefully consider the risks and benefits. Future research should systematically investigate the potential for undue inducement among those who make the decision to enroll relatively quickly.^27,28^

### Limitations

This study has limitations. Participation in SHIFT was time intensive, with an involved study intervention. Time and logistics are known barriers to engagement in this type of family-based behavioral intervention,^29^ and these factors may have played a larger role than usual in this enrollment decision. The physical risks associated with SHIFT were also relatively low; individuals might take longer to decide about higher-risk research. SHIFT also engaged mostly in passive recruitment through flyer distribution; parents needed to proactively contact the study staff to start the enrollment process. Studies that do not share these characteristics might have different decision-making patterns.

Our sample size was limited to the pool of SHIFT enrollees and decliners who agreed to provide their contact information. More participants than decliners completed our survey, resulting in participation bias. Twenty-two individuals declined to allow their information to be shared, and 24 decliners could not be asked owing to losing contact during the process. Therefore, our sample of decliners may also be biased, as those who had negative interactions with the study team or other reasons for declining may have chosen not to provide their contact information for this survey.

In addition, our respondents were largely women, non-Hispanic White, and highly educated. Further work is needed to understand decision-making among a more diverse group, especially as more attention is being paid to how to improve informed consent for traditionally underrepresented populations.^30,31^

## Conclusions

The findings of this survey study suggest that many parents make research decisions after receiving study information and engaging with staff but before review of the consent form. This result offers one possible explanation for why efforts to improve consent forms have had limited effects on decision-making and highlights the need for regulatory review and intervention development to focus on the early engagement of prospective participants. This result is especially notable given the pediatric setting, in which parents may be more deliberate about decision-making than if they were making decisions for themselves and may therefore offer a conservative estimate of how early in the process research decisions are sometimes made, with relatively limited information about the study. Future research should examine whether decisions in different research settings, such as studies with higher risk, related to acute conditions, or in adult populations, similarly occur with minimal, if any, reliance on the consent form.

## Notes

Supplement. **eAppendix.** Parent Survey
