# Supplementary material for: Assessing Parent Decisions About Child Participation in a Behavioral Health Intervention Study and Utility of Informed Consent Forms
Source: JAMA Netw Open. 2020 Jul 31;3(7):e209296. doi: 10.1001/jamanetworkopen.2020.9296 (PMC7395235; doi:10.1001/jamanetworkopen.2020.9296)
Supplement: Supplement. — eAppendix. Parent Survey [file jamanetwopen-3-e209296-s001.pdf]

Supplementary Online Content

Kraft SA, Porter KM, Duenas DM, et al. Assessing parent decisions about child participation in a behavioral health intervention study and utility of informed consent forms. *JAMA Netw Open*. 2020;3(7):e209296. doi:10.1001/jamanetworkopen.2020.9296

eAppendix. Parent Survey

This supplementary material has been provided by the authors to give readers additional information about their work.

© 2020 Kraft SA et al. *JAMA Network Open.*

eAppendix. Parent Survey

Understanding Research Decisions

Thanks for agreeing to do this survey. We are asking you questions to help us understand how and when people decide whether to be in a research study. We will ask how you decided whether or not to join the SHIFT study.

We are working with the SHIFT study team, but we are not part of the SHIFT team. Your answers will be kept confidential and will not affect your participation in the SHIFT study, your healthcare, or your child's healthcare.

You're free to skip questions or to stop answering the survey at any time. It should take about 10 minutes to complete this survey.

First, we have some questions about the SHIFT study.

How did your family first hear about the SHIFT study?

- o A flyer in the mail
- o My doctor or my child's doctor
- o A website
- o Newspaper or TV
- o Social media, such as Facebook or Twitter
- o A friend
- o Other → How did your family hear about it? _______________

When you first heard about the SHIFT study, what did you think?

- o It sounded like a good idea to be part of it
- o It sounded like a bad idea to be part of it
- o No opinion
- o Don't remember

Other than contacting the study team, did you do anything else to try to learn more about the study after you first heard about it? (For example, by talking with a friend or doctor, looking at the study website, or searching the internet, or through some other source?)

- o Yes → What did you do? _______________
- o No

How much did you like the member of the SHIFT study team who first talked with you and/or your family member over the phone?

o Not at all   o Not much   o Somewhat   o Very much

© 2020 Kraft SA et al. *JAMA Network Open.*

How much did you trust the member of the SHIFT study team who first talked with you and/or your family member over the phone?

o Not at all	o Not much	o Somewhat	o Very much

What did you like about the study right after you and/or your family member had the first conversation with the study team? (Choose all that apply)

- o I hoped it would help me and/or my child
- o I wanted to learn from it
- o I wanted to help develop new knowledge about childhood obesity and advance science
- o I wanted to help the community of parents and children with childhood obesity
- o I was interested in the financial incentives
- o The risks of the study seemed low
- o Other → Please specify: _______________
- o I did not like anything about the study
- o Don't remember

What didn't you like about the study right after you and/or your family member had the first conversation with the study team? (Choose all that apply)

- o I was not sure it would help me and/or my child
- o I was worried it could harm me and/or my child
- o I thought they would treat me and/or my child like a guinea pig
- o The timing, location, or transportation to the site was inconvenient
- o The treatment required too much time/effort
- o The monetary incentives were not enough to justify being in it
- o Other → Please specify: _______________
- o I did not dislike anything about the study
- o Don't remember

Now please think about the SHIFT orientation visit, where you may have been shown a presentation about the SHIFT study.

Did you attend a SHIFT study orientation visit?

- o Yes
- o No
- o Don't remember

When you arrived at the orientation visit, how certain were you that you would say yes to being in the study?

o Not at all certain	o Not certain	o Somewhat certain	o Very certain

© 2020 Kraft SA et al. *JAMA Network Open.*

How much attention did you pay during the presentation about the SHFIT study at your orientation visit?

- ○ None at all ○ Not much ○ Some ○ Very much

Did the presentation teach you anything new about the study?

- ○ Yes
- ○ No
- ○ Don't remember

How did the presentation affect your interest in participating in the SHIFT study?

- ○ It raised some concerns or made me less interested ○ No change ○ It made me more interested

How much did you like the members of the SHIFT study team who talked with you at the orientation visit?

- ○ Not at all ○ Not much ○ Somewhat ○ Very much

How much did you trust the members of the SHIFT study team who talked with you at the orientation visit?

- ○ Not at all ○ Not much ○ Somewhat ○ Very much

Now, please think about the consent process, where you may have been given a consent form to read.

Were you given a consent form to read?

- ○ Yes
- ○ No
- ○ Don't remember

How much of the consent form did you read?

- ○ None of it ○ Parts of it ○ Most of it ○ All of it

How carefully did you read the consent form?

- ○ Not at all carefully ○ Not carefully ○ Somewhat carefully ○ Very carefully

© 2020 Kraft SA et al. *JAMA Network Open.*

How helpful was the consent form?

- Not at all helpful → What made it unhelpful? __________ __________
- Not very helpful → What made it unhelpful? __________ __________
- Somewhat helpful → What made it helpful? __________ __________
- Very helpful → What made it helpful? __________ __________

Did reading the consent form teach you anything new about the study?

- Yes
- No
- Don't remember

Now, please think about when you made your decision whether or not to join the SHIFT study.

Did you decide to join the SHIFT study?

- Yes
- No

When would you say you made your decision about joining or not joining the study?

- Almost right after I first heard about it
- Not right away, but before I talked with the study team
- After I talked with the study team about the study on the phone
- After the presentation at the orientation visit
- After the study team answered questions at the orientation visit
- After the orientation visit but before I received the consent form
- When I first received the consent form
- After I read the consent form
- Other → Please specify: ________________

What was most important in helping you make your decision?

- Internet search
- Talking with my spouse or a trusted friend
- Talking with my child
- Talking with my doctor
- Talking with the research staff
- The study flyer
- The informed consent document
- Other → Please specify: ________________

© 2020 Kraft SA et al. *JAMA Network Open.*

Is there anything that you wish had been different that could have helped you make this decision? ____

Finally, we would like some information about you. This information is for comparison purposes only, and your answers will be kept confidential.

Have you ever been in a research study before?

- o Yes → How many research studies have you been in? _______________
- o No
- o Don't remember
- o Prefer not to answer

What is your highest education level?

- o Some high school
- o Graduated high school
- o Some college
- o Graduated college
- o Completed trade/technical/vocational training
- o Some postgraduate work
- o Graduate/professional degree or higher
- o Prefer not to answer

What was your total household income last year?

- o Less than $10,000
- o $10,000 to $29,999
- o $30,000 to $69,999
- o $70,000 to $99,999
- o $100,000 to $149,999
- o $150,000 or more
- o Don't know
- o Prefer not to answer

What is your gender?

- o Male
- o Female
- o Transgender
- o Other → Please specify: _______________
- o Prefer not to answer

© 2020 Kraft SA et al. *JAMA Network Open.*

What is your marital status?

- Married
- Widowed
- Divorced
- Separated
- Never married
- Prefer not to answer

What is your age?

- 18-25
- 26-49
- 50-64
- 65 or older
- Prefer not to answer

How would you describe your race or ethnicity? (Choose all that apply)

- White
- Black or African American
- Hispanic or Latino/a
- Native American
- Asian American
- Native Hawaiian or Pacific Islander
- Other → Please specify: _______________
- Don't know
- Prefer not to answer

Thank you for taking the time to participate in our survey. We truly value the information you have provided, and hope that our survey will help researchers to understand when people make decisions about research and how to provide them with the information and support that they need.

© 2020 Kraft SA et al. *JAMA Network Open.*
